# Inhibition Controls Asynchronous States of Neuronal Networks

**DOI:** 10.3389/fnsyn.2016.00011

**Published:** 2016-05-12

**Authors:** Mario Treviño

**Affiliations:** Instituto de Neurociencias, Universidad de GuadalajaraGuadalajara, Mexico

**Keywords:** oscillations, network, cortex, inhibition, interneuron, synchrony

## Abstract

Computations in cortical circuits require action potentials from excitatory and inhibitory neurons. In this mini-review, I first provide a quick overview of findings that indicate that GABAergic neurons play a fundamental role in coordinating spikes and generating synchronized network activity. Next, I argue that these observations helped popularize the notion that network oscillations require a high degree of spike correlations among interneurons which, in turn, produce synchronous inhibition of the local microcircuit. The aim of this text is to discuss some recent experimental and computational findings that support a complementary view: one in which interneurons participate actively in producing *asynchronous* states in cortical networks. This requires a proper mixture of shared excitation and inhibition leading to asynchronous activity between neighboring cells. Such contribution from interneurons would be extremely important because it would tend to reduce the spike correlation between neighboring pyramidal cells, a drop in redundancy that could enhance the information-processing capacity of neural networks.

## Introduction

Glutamatergic (excitatory) and GABAergic (inhibitory) neurons can be classified on the basis of their morphology and electrophysiological properties, as well as on the neuromodulatory receptors expressed on their membranes. Pyramidal neurons (PyrCs) are the principal type of excitatory cells in cortex. Each one receives approximately 10^4^ synaptic inputs, of which approximately 75% are excitatory and 25% inhibitory (Amaral and Witter, 1989; Ishizuka et al., 1995). The excitatory synapses on PyrCs are distributed along their dendrites, whereas the inhibitory synapses are located throughout their dendritic tree, the soma, and the initial axon segment. Thus, in PyrCs, excitatory and inhibitory inputs co-exist on the peripheral dendritic tree, while the proximal segments receive only inhibition (Pouille et al., 2013).

Although far less numerous, the GABAergic interneurons (INTs) are exceptionally diverse in terms of their biochemical, biophysical and morphological properties, as well as in their connectivity with other neurons (Freund and Buzsáki, 1996; Kawaguchi and Kubota, 1997; Somogyi and Klausberger, 2005). Probably due to the high metabolic costs involved in building and maintaining long-range axonal projections, the wiring of most INTs is local. An apparent inverse relationship between potential synaptic contacts from interneurons and the distance of their axonal projection supports this idea (Gupta et al., 2000; Buzsáki et al., 2004; Ferreira et al., 2014). One effect of this arrangement is that neighboring PyrCs and INTs share some of their synaptic inputs and process similar information.

Neurons in the brain receive concurrent excitatory and inhibitory inputs. Depending on the driving force, a synaptic response can produce either a depolarization, a hyperpolarization or simply no change in the membrane potential of the postsynaptic cell. Increasing the net synaptic conductance lowers the input resistance (due to more simultaneous inputs), reducing the voltage gradient generated by additional currents and the membrane time constant of the cell. In consequence, synaptic inhibition can decrease the amount of membrane depolarization in two complementary ways: by producing a hyperpolarizing current (i.e., a “subtractive” effect), or by increasing the global conductance of the cell by acting in such a way as to divide all the synaptic currents (i.e., “shunting inhibition”, also known as “silent inhibition”; Koch et al., 1990; Borg-Graham et al., 1998; Treviño and Gutiérrez, 2005; Silver, 2010; Treviño et al., 2011). Theoretical work supports this notion and predicts that inhibition will have a “divisive” effect on the postsynaptic potentials if conductance change is large and located close to the soma, but will produce a “subtractive” effect if the changes in input conductance are small and spatially-distributed (Silver, 2010). For all these reasons, INTs are key players in controlling the dynamics of recurrent excitatory circuits at various spatial and temporal scales. Perisomatic inhibition tightly regulates the spike timing of postsynaptic cells and is able to synchronize entire neural ensembles (Freund and Buzsáki, 1996; Glickfeld and Scanziani, 2006; Mann and Paulsen, 2007; Silberberg and Markram, 2007).

## Building Principles of Cortical Circuits

**Figure 1:**
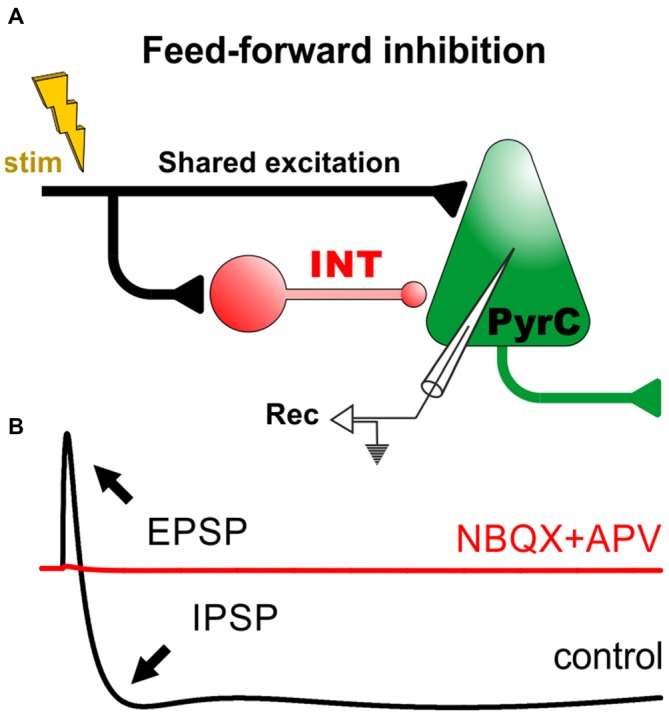
**Feed-forward inhibitory circuits and the input/output function of cortical pyramidal cells. (A)** Diagram of a minimal circuit with feed-forward inhibition (FFI). The circuit consists of an excitatory projection (in black) that drives an interneuron (INT) and a pyramidal cell (PyrC). The INT makes a synaptic contact with the PyrC. **(B)** The lower panel displays a cartoon of a prototypical intracellular recording from the PyrC in control conditions (black trace). For example, in the hippocampus and cortex, activation of the excitatory projection generally produces a monosynaptic excitatory postsynaptic potential (EPSP), followed by a di-synaptic fast inhibitory postsynaptic potential (IPSP; GABA_A_-receptor-dependent) and a slow IPSP (GABA_B_-receptor-dependent). In these conditions, the addition of ionotropic glutamate receptor antagonists (NBQX+APV) blocks all synaptic responses, revealing the polysynaptic nature of FFI in these circuits (red trace; McCormick, 1992; Treviño and Gutiérrez, 2005; Treviño et al., 2007, 2011).

The complex patterns of excitatory and inhibitory connections in the brain provide the structural basis for a rich repertoire of network activities. Cortical INTs can perform both feed-forward and feed-back inhibitory actions based on their input activity and their sensitivity to neuromodulators (Salgado et al., 2012, 2016). In feed-forward inhibition (FFI), an INT outputs its inhibitory signal to a PyrC that receives shared excitation (Figure 1A). Typical *in vitro* recordings performed from PyrCs responding to stimulation of afferent fibers show a compound synaptic response that consists of an excitatory postsynaptic potential (EPSP) followed by an inhibitory postsynaptic potential (IPSP) mediated by the activation of GABA_A_ and, sometimes, GABA_B_ receptors (Figure 1B). This built-in structural inequality sharpens the EPSP by narrowing the temporal “window of opportunity” for action potential generation, while also reducing the overall number of action potentials and limiting the temporal summation of EPSPs in the PyrCs (McCormick, 1992; Pouille and Scanziani, 2001; Maccaferri and Dingledine, 2002; Lawrence and McBain, 2003; Gabernet et al., 2005; Higley and Contreras, 2006; Ferrante et al., 2009; Torborg et al., 2010). The nature and strength of inhibition regulate the propagation speed of excitatory activity through cortical circuits (Trevelyan et al., 2007; Moldakarimov et al., 2015).

The generation of an action potential in cortical neurons is the result of complex spatio-temporal interactions between simultaneous synaptic inputs (Treviño et al., 2011). The precise relationship between synaptic excitation and inhibition (i.e., the E/I ratio) determines fundamental cortical operations such as feature selectivity and gain (Haider and McCormick, 2009; Isaacson and Scanziani, 2011). Interestingly, despite fluctuating network activity levels, PyrCs in the cortex show quite stable averaged E/I ratios over time. This cortical “E/I balance” is achieved because inhibition increases proportionally with excitation, mainly through an enhanced recruitment of INTs (Anderson et al., 2000; Shu et al., 2003; Haider et al., 2006; Okun and Lampl, 2008; Xue et al., 2014). Yet, to complete this concept: some short-term plastic changes in the strength of synaptic transmission can also lead to small but progressive changes in the E/I balance. In the cerebral cortex, for example, both excitation and inhibition on PyrCs undergo short-term depression during repetitive electrical stimulation, but inhibition has a greater depressive effect, increasing the E/I ratio. This, in turn, leads to a progressively longer temporal window for EPSP summation and a decrease in spike-timing precision. Similarly, in the hippocampus, a single action potential in a dentate granule cell evokes a net inhibitory potential in CA3 PyrCs (Figure 1B), but this input signal can lead to an excitatory output if the stimulation frequency is increased (Gabernet et al., 2005; Treviño et al., 2007, 2011). The process responsible for this switch involves the facilitation of excitatory responses coupled with a rapid depression of inhibitory transmission (Mori et al., 2004; Szabadics and Soltesz, 2009). A last example: in hippocampal CA1, feed-forward excitatory synaptic strength onto fast-spiking basket cells remains quite constant, whereas monosynaptic connections between fast-spiking basket cells and PyrCs are depressed during repetitive stimulation, resulting in an overall depression of the inhibitory drive onto PyrCs. Thus, the E/I balance can be regulated in a frequency-dependent manner by short-term changes in synaptic transmission (Galarreta and Hestrin, 1998; Gabernet et al., 2005; Glickfeld and Scanziani, 2006).

## The Input-Output Transfer Function of Cortical Neurons

The probability that a neuron will fire an action potential in response to afferent stimuli is described by its input-output (I/O) function. The I/O function depends on the passive and active electrical properties of the cell, but also on the dendritic morphology and on the E/I balance. Mathematically, this function can be represented by a logistic curve: the neuron is nearly silent at low input rates, but increases its firing output with a given slope (also referred to as the “gain control”) before finally reaching a plateau at saturation input rates (Llinás, 1988; Bartesaghi et al., 2006; Campanac and Debanne, 2008; Pouille et al., 2013).

Typically, the all-or-none properties of axonal spiking, together with the presence of active dendritic properties, yield a steep I/O sigmoid with a very limited operative range for input stimuli. However, because neurons are regularly subjected to an intense ongoing synaptic bombardment *in vivo*, this leads to an actual I/O function with a smaller gain. In other words, the combination of variable “background” synaptic noise with the intrinsic properties of the cells renders an I/O relationship with a broader dynamic range (for input signals), mixing single-spike and burst responses at multiple membrane potentials (Borg-Graham et al., 1998; Paré et al., 1998; Wolfart et al., 2005; Monier et al., 2008). Mechanistically, transformations of the I/O relationship can occur because the voltage fluctuations allow synaptic inputs to cross the action potential threshold more frequently, even when the average membrane potential is well below the spike threshold. Interestingly, such high levels of voltage noise fluctuations can only be achieved during uncorrelated (asynchronous) inputs, when excitatory and inhibitory synaptic conductances are, on average, well-balanced (Okun and Lampl, 2008; Petersen and Crochet, 2013).

## Inhibition Controls Asynchronous States of Neuronal Networks

**Figure 2:**
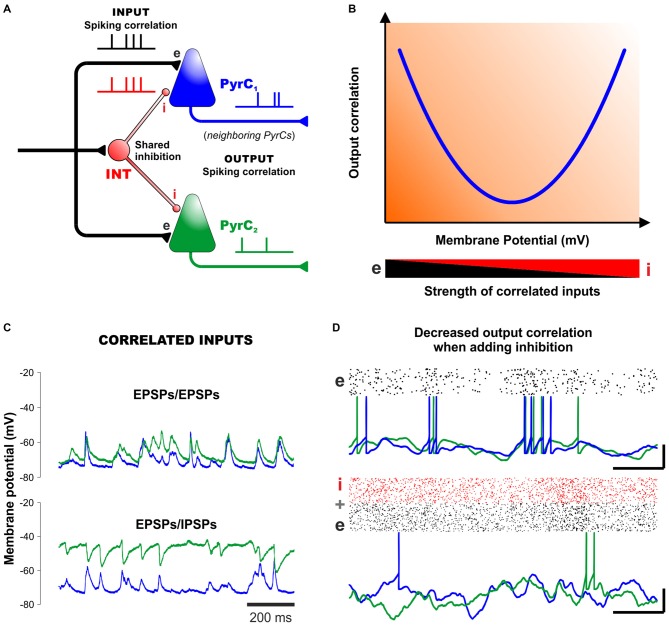
**Correlated inhibition and excitation cancel each other out to create asynchronous network states. (A)** Diagram of a minimal cortical circuit depicting how shared excitation and inhibition can produce correlated excitatory and inhibitory responses in two neighboring pyramidal cells. **(B)** Schematic outline of the output correlation of synaptic currents (and also output spikes) between neighboring cells observed under different strengths of excitation and inhibition (*v.gr*. using different membrane potentials, represented in the x-axis). This V-shaped relationship, predicted theoretically (Renart et al., 2010) and confirmed experimentally (Sippy and Yuste, 2013) suggests that excitatory and inhibitory inputs into neighboring PyrCs are indeed correlated. The resulting membrane currents and spike output become less correlated at physiological membrane potentials by mutual cancellation. **(C)** Intracellular recordings from neighboring PyrCs near the reversal potential of inhibition (−75 mV, top). In the panel below, one cell is injected with a small positive current to reveal the inhibitory potentials (with QX-314 added to the pipette to prevent firing). Positive deflections of the membrane potential in the upper panel reflect mostly excitatory synaptic currents, whereas the negative deflections in the depolarized trace (green trace) reflect inhibitory currents. Note how the excitatory and inhibitory inputs are highly-correlated in neighboring PyrCs. **(D)** Computer simulations show that inhibition (lower panel) decreases the correlation observed in networks based on excitatory cells only (upper panel). Scalebars (15 mV, 50 ms). Panels **(B–D)** modified, with permission, from Okun and Lampl (2008) and Renart et al. (2010).

Evidence from *in vitro* and *in vivo* recordings indicates that the activity of INTs plays a major role in coordinating spikes and generating oscillatory network activity (Freund and Buzsáki, 1996; Bartos et al., 2007; Treviño et al., 2007). By counterbalancing excitatory inputs in a temporally precise way, correlated inhibition can orchestrate action potential timing in multiple postsynaptic neurons. This is crucial for general cortical functioning and also for inducing synaptic plasticity (Kwag and Paulsen, 2009). Contrary to this view, however, recent experimental evidence reveals that neural networks, both *in vitro* and *in vivo*, generally display asynchronous states with low spiking correlations among cortical neurons including INTs (Okun and Lampl, 2008; Petersen and Crochet, 2013; Sippy and Yuste, 2013). One candidate mechanism to explain the low correlated spiking output is that presynaptic excitatory and inhibitory (phasic) inputs are themselves correlated, shunting the membranes of postsynaptic cells. Some authors have interpreted the high correlations in IPSCs observed in neighboring PyrCs as an indication of a high correlation in the spiking activity of presynaptic INTs (Hasenstaub et al., 2005). However, recent experiments indicate that correlations in IPSCs can be easily explained by the fact that nearby cells share some of their inhibitory input (Figure 2A). In other words, this means that inhibition can indeed be locally coherent, but not due to synchrony among INTs, but simply because PyrCs share common presynaptic inputs from INTs. Here, importantly, inhibition is globally no more correlated than excitation (Okun and Lampl, 2008; Sippy and Yuste, 2013).

In their theoretical model of de-correlated networks, Renart et al. (2010) proposed that shared excitatory and inhibitory input correlations could indeed explain the low levels of spiking (output) correlation between pairs of PyrCs, as observed in dozens of *in vivo* experiments (Figure 2A). To test this model, Sippy and Yuste (2013) performed dual voltage-clamp recordings in PyrCs from brain slices during thalamically-triggered UP states at different holding potentials. This strategy changed the driving force of inhibition and excitation (at least in the perisomatic region) and provided control of their relative synaptic efficacy. In agreement with the model, the intracellular recordings revealed a V-shaped relationship between the correlation of membrane currents of nearby PyrCs and their membrane potential (Figure 2B). That is, the correlation between synaptic currents was highest at −70 mV and +0 mV, where excitation and inhibition are relatively isolated, whereas at intermediate potentials, the net effect of the synchronous excitatory and inhibitory currents lead to a lower output correlation between the membranes of nearby PyrCs (Sippy and Yuste, 2013; see also Hasenstaub et al., 2005). Thus, a proper mixture of excitation and inhibition leads to a state of relative asynchrony between neighboring cells (Figures 2A,B). Certainly, in this explanatory framework, inhibition and excitation must be correlated in postsynaptic membranes (Renart et al., 2010; Sippy and Yuste, 2013).

Further support to these ideas: *in vivo* experiments by Okun and Lampl (2008) have shown that, during spontaneous and sensory-evoked activities, neighboring cortical cells display highly-correlated: (i) EPSPs/EPSPs (Figure 2C, upper panel); (ii) IPSPs/IPSPs; and (iii) EPSPs/IPSPs (Figure 2C, lower panel). Moreover, the cross-correlations of the EPSPs and IPSPs from neighboring cells peaked at a positive delay of ~3 ms. This indicates that, on average, inhibition lags behind excitation by several milliseconds. Such tight coupling is consistent with the hypothesis that inhibition controls the integration time window of excitation, enabling neurons to operate as coincidence detectors. In other words, because nearby cortical neurons receive highly-similar excitatory inputs, FFI might indeed constitute a robust mechanism for de-correlating network activity (Figure 2D).

Assuming that FFI accounts for the strong correlations between excitatory and inhibitory inputs into PyrCs, it would be natural to ask whether the correlation state of a network could be sensitive to (or manipulated by) changes in the inhibitory “tone” in cortical networks. This has been confirmed experimentally: substantial increases in network synchrony occur both *in vitro* and *in vivo* when using pharmacological agonists that reduce the probability of GABA release, and also with antagonists that block GABAergic conductances (Cohen and Miles, 2000; Treviño et al., 2007; Petersen and Crochet, 2013). Similarly, a partial blockage of GABAergic receptors can produce an increase in pair-wise correlations of EPSCs between PyrCs, confirming that IPSCs participate in de-correlating output spikes (Sippy and Yuste, 2013). Therefore, reducing the GABA function increases network synchrony *in vitro*, whereas adding INTs into simulated networks *in silico* decreases their output correlation (Figure 2D).

## Possible Computational Benefit from Asynchronous Activity

Neural activity can represent information as a firing rate, as correlations in spike-timing, or as a complex combination of both (Silver, 2010; Luczak et al., 2015). Many researchers believe that networks can display a rich repertoire of synchronous and asynchronous states and that they can switch between them by adjusting the E/I balance. Experimental measurements *in vivo* corroborate this view, and theoretical studies suggest that networks can self-organize to reach such balanced states in which different neurons emit action potentials asynchronously (van Vreeswijk and Sompolinsky, 1996; Brunel, 2000; Renart et al., 2010). Two fundamentally different types of asynchronous activity have been distinguished (Ostojic, 2014). When synaptic couplings between neurons are weak, the network at rest can operate in an “homogenous asynchronous state”, a regime well-suited for transmitting information about the firing rate of external inputs. This is because neurons in the network will change their mean firing rate in proportion to the input. In contrast, for strong couplings, a network at rest displays rich internal dynamics, in which the firing rates of individual neurons fluctuate strongly, both in time and across neurons. In this scenario, the internal state of the network is such that repeated presentations of the same external stimulus leads to very different responses (i.e., the input is dynamically transformed). Certainly, this variability in the population degrades the transmission of information but provides a rich substrate for a nonlinear processing of the stimuli (Ostojic, 2014).

Neuronal responses are typically variable in the sense that the number and timing of the spikes in response to the same stimulus is never identical among trials (Lee et al., 1998; Kilgard and Merzenich, 1999; Ringach et al., 2002; Chelaru and Dragoi, 2008; Moreno-Bote et al., 2014). The responses of nearby neurons located within the same “functional column” in the cortex, which are thought to encode the “same stimulus property”, exhibit a high degree of heterogeneity. Yet, interestingly, the trial-by-trial variability in neuronal responses is not independent, but exhibits correlations (Zohary et al., 1994; Lee et al., 1998; Ringach et al., 2002; Chelaru and Dragoi, 2008). One possibility could be that population coding depends not only on the response properties of the cells but also on the distribution of neuronal correlations across the network (Abbott and Dayan, 1999; Pouget et al., 2000; Sompolinsky et al., 2001; Luczak et al., 2015). And in this respect, the high variability of intrinsic response properties of individual cells may change the structure of neuronal correlations thereby improving the information encoded in the population activity (Chelaru and Dragoi, 2008). This proposal predicts that the most energy-efficient codes for representing information would be sparse, as observed in multiple *in vivo* preparations (Petersen and Crochet, 2013). Under a sparse coding regime, a neuron could act as a coincidence detector of temporally-correlated inputs. Furthermore, computational strategies used by the brain should depend on the amount of information that can be stored in population activity. *In vivo*, noise correlations tend to be positive and proportional to the similarity in tuning properties, and are thought to limit information. It would be thus natural to think that de-correlating neuronal activity could increase the information capacity of the network (Abbott and Dayan, 1999; Pouget et al., 2000; Sompolinsky et al., 2001; Seriès et al., 2004). In other words, because information usually decreases as correlations increase, it could be advantageous if the brain were to possess a mechanism to de-correlate neural activity, either through a passive process such as balancing or synchronizing excitation and inhibition, or an active one such as attention (Cohen and Maunsell, 2009; Mitchell et al., 2009; Renart et al., 2010).

## Concluding Remarks

Inhibition is generally conceptualized as a mechanism that restricts the probability of action potential generation by reorganizing spikes in multiple cells and promoting network oscillations (Bartos et al., 2007). Here, I have discussed how inhibition can also promote and control the generation of asynchronous states. A proposal is that depending on the spatiotemporal dynamics of synaptic inputs, inhibition could act as a correlating or de-correlating agent that controls network synchrony. Signal integration of postsynaptic cells is transformed through shared inhibition, affecting their I/O transfer function and de-correlating spikes in neighboring cells which might increase the network’s storage-capacity (but see Moreno-Bote et al., 2014).

Spontaneous spiking by most cortical neurons *in vivo* and *in vitro* is generally asynchronous and infrequent (usually <1 Hz; Renart et al., 2010; Petersen and Crochet, 2013) but, intuitively, such sporadic activity should eventually reach a point of complete inactivity because it cannot sufficiently depolarize postsynaptic neurons to reach their spike threshold. How cortical networks are able to maintain asynchronous and infrequent activity is unclear. Maybe spikes result from cell-intrinsic—i.e., non-synaptically driven—processes: some studies suggest that certain brain areas can generate discharges in the absence of (sensory) evoked synaptic inputs (e.g., Cohen and Miles, 2000). Different levels of neuromodulators, or different attentional or arousal levels could also regulate the intrinsic conductances and the firing and excitability of neurons as well as their transition into desynchronized UP states *in vivo* (Destexhe et al., 2003; Wladyka and Kunze, 2006; Constantinople and Bruno, 2011). Perhaps both intrinsic cellular properties and shared synaptic interactions contribute to the asynchronous state of cortical networks. If so, fine-tuning of the “E/I balance” may be a key mechanism for modulating spike output correlations and transitions among brain states.

## Author Contributions

MT conceived and wrote the article.

## Funding

Research in my laboratory was funded by grants from the Consejo Nacional de Ciencia y Tecnología (CONACyT; 220862, 251406).

## Conflict of Interest Statement

The author declares that the research was conducted in the absence of any commercial or financial relationships that could be construed as a potential conflict of interest. The reviewer AT and handling Editor MC declared their shared affiliation, and the handling Editor states that the process nevertheless met the standards of a fair and objective review.
